# Framework and indicator testing protocol for developing and piloting quality indicators for the UK quality and outcomes framework

**DOI:** 10.1186/1471-2296-12-85

**Published:** 2011-08-10

**Authors:** Stephen M Campbell, Evangelos Kontopantelis, Kerin Hannon, Martyn Burke, Annette Barber, Helen E Lester

**Affiliations:** 1 Health Sciences Research Group - Primary Care, University of Manchester, 7th Floor Williamson Building, Oxford Road, Manchester, M13 9PL. UK; 2 York Health Economics Consortium, University of York, York., UK

## Abstract

**Background:**

Quality measures should be subjected to a testing protocol before being used in practice using key attributes such as acceptability, feasibility and reliability, as well as identifying issues derived from actual implementation and unintended consequences. We describe the methodologies and results of an indicator testing protocol (ITP) using data from proposed quality indicators for the United Kingdom Quality and Outcomes Framework (QOF).

**Methods:**

The indicator testing protocol involved a multi-step and methodological process: 1) The RAND/UCLA Appropriateness Method, to test clarity and necessity, 2) data extraction from patients' medical records, to test technical feasibility and reliability, 3) diaries, to test workload, 4) cost-effectiveness modelling, and 5) semi-structured interviews, to test acceptability, implementation issues and unintended consequences. Testing was conducted in a sample of representative family practices in England. These methods were combined into an overall recommendation for each tested indicator.

**Results:**

Using an indicator testing protocol as part of piloting was seen as a valuable way of testing potential indicators in 'real world' settings. Pilot 1 (October 2009-March 2010) involved thirteen indicators across six clinical domains and twelve indicators passed the indicator testing protocol. However, the indicator testing protocol identified a number of implementation issues and unintended consequences that can be rectified or removed prior to national roll out. A palliative care indicator is used as an exemplar of the value of piloting using a multiple attribute indicator testing protocol - while technically feasible and reliable, it was unacceptable to practice staff and raised concerns about potentially causing actual patient harm.

**Conclusions:**

This indicator testing protocol is one example of a protocol that may be useful in assessing potential quality indicators when adapted to specific country health care settings and may be of use to policy-makers and researchers worldwide to test the likely effect of implementing indicators prior to roll out. It builds on and codifies existing literature and other testing protocols to create a field testing methodology that can be used to produce country specific quality indicators for pay-for-performance or quality improvement schemes.

## Background

Quality measures are increasingly used internationally to measure the quality of health care. In many cases, but not all, these are used as part of pay-for-performance schemes [1-4]. Any quality assessment measure must adhere to certain key attributes [5-12]. These include a clear definition and purpose, their acceptability to assessors and those being assessed, needs assessment, clinical feasibility and relevance, sensitivity to change, potential for improvement, discrimination/variance, technical feasibility and reliability of data extraction, an understanding of how they will be implemented and validity (including evidence base and addressing unintended consequences). Measures must also be 100% under the direct control of those being assessed (attribution or controllability) or lesser control must be reflected in the assessment. Quality measures, in their development, implementation and in the interpretation of the results, should be subjected to a testing protocol, whereby indicators are assessed against such attributes.

Testing protocols have been developed, mostly for use in the United States; for example, the Physician Consortium for Performance Improvement (PCPI) of the American Medical Association (AMA) [9] or the National Committee for Quality Assurance (NCQA), which develops the Health Plan Employer Data and Information Set (HEDIS) [6]. Both the PCPI [9] and NCQA [6] use detailed measurement methodologies and in the case of NCQA, the subsequent HEDIS measures are used by more than 90 percent of health plans in the United States. Piloting has also been routinely included as part of Veterans Administration indicator development method [13].

In 2004, the United Kingdom government introduced the Quality and Outcomes Framework (QOF), a pay-for-performance scheme, which consists of clinical and organisational quality indicators [8]. The original 2004 QOF indicators, and all subsequent changes to indicators, were introduced without piloting. In 2009 a new way of developing clinical indicators for QOF was introduced, led by the National Institute for Health and Clinical Excellence (NICE) [14]. NICE now prioritise areas for clinical quality indicator development, based on national guidelines as source material.

The development and piloting of potential QOF indicators is contracted to an independent external contractor, led by the authors, which has two main roles. Our first role is to review existing QOF indicators and recommend whether indicators should remain or be removed or revised. There has been little work at an international level on this issue and we have developed and described a set of underpinning principles for indicator replacement and key issues that need to be considered by any organization or country planning to remove indicators from a clinical performance framework [15,16].

Our second role is to develop and pilot new indicators for potential inclusion in QOF. Prior to 2009, none of the 146 indicators in the original QOF had been piloted. Practical problems with implementation subsequently occurred, some of which took up to 6 months to correct during 'live QOF'. Since over 1 billion pounds is attached to achieving these indicators on an annual basis, this is less than ideal. The advocacy of piloting new indicators in the UK, is not new [17,18]. Nor is the awareness of the importance of obtaining baseline data before implementation [19]. However, prior to 2009 there was no published indicator testing protocol for the development and piloting of quality indicators in the UK.

### Objective

This paper describes the multiple stage development and piloting processes of the indicator testing protocol that we developed as part of the process of piloting indicators for the UK QOF. It also presents, the results from the first QOF pilot, and shows how this testing protocol compares to and builds upon existing literature. The testing protocol described is one example of a protocol that may be useful in assessing potential quality indicators when adapted to specific country health care settings is and may be of use to policy-makers and researchers worldwide to test the likely effect of implementing indicators prior to roll out.

## Methods

**Figure 1:**
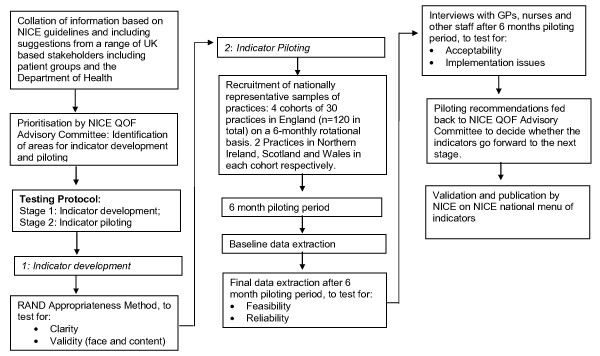
**Flow diagram of the Indicator Testing Protocol**.

The indicator testing protocol consists of two stages: indicator development and indicator testing (see Figure 1).

### Stage 1: Indicator development

Clinical conditions/issues recommended for indicator development, which are supported by a NICE/SIGN (Scottish Intercollegiate Guidelines Network) guideline, are suggested for development by a NICE Advisory Committee that sits twice yearly in June and December (http://www.nice.org.uk/aboutnice/qof/PrimaryCareQOFIndicatorAdvisoryCommittee.jsp) (Figure 1). For each suggested area, we (the external contractor group and authors of this paper) then develop a set of initial indicator 'sets'. Each set represents a coherent group of indicators in the same clinical domain (e.g. diabetes) that avoid duplication and address specifically defined aspects of quality of care. The key to successful indicator development is to precisely define the meaning and specifications of an indicator so that what is being assessed is clear and the assessment of quality is attributable directly to that indicator [5,6,20]. The presence of such precision of meaning and purpose facilitates subsequent analyses of other indicator attributes such as technical feasibility.

We use the RAND/UCLA Appropriateness Method to rate the initial sets of indicators. This combines available scientific evidence with the collective judgement of experts by deriving a consensus opinion from a group, with individual opinions aggregated into refined aggregated opinion [5,21,22]. The aim is to identify indicators that, if implemented, are likely to provide net benefit to patients and improve patient outcomes. Panel composition is the major determinant of final ratings [22]. We appoint a 10 member panel of front line GPs and GPs with a special clinical interest in the indicator areas under discussion.

The RAND/UCLA Appropriateness Method involves two rounds of rating, the first conducted by post and the second at a face-to-face meeting [21]. Panel members are sent a summary of the evidence and the list of indicators together with an instruction sheet and set of definitions for all the terms used. Indicators that go forward to piloting must adhere to the RAND/UCLA Appropriateness Method definition of consensus (overall panel median of 7-9) with agreement (80% of ratings within the 3-point tertile of the overall median) for both clarity and necessity [5,21].

### Stage 2: Indicator testing

As part of the indicator testing process, mixed methodologies are used to test particular indicator attributes (Table 1), based on an overall indicator testing protocol (Appendix 1).

**Table 1:** Indicator testing protocol feedback form

Attribute	Summary of method
Clarity	• RAND Appropriateness Method ratings *

Necessity	• RAND Appropriateness Method ratings *

Acceptability	• Risks, issues, relative impact, and uncertainties (interviews with practice staff)

Feasibility	• 'Technically feasiblility' in current family practice systems and whether supported by current methods of data extraction for QOF (data extraction in all family practice clinical systems)

Reliability	• Reproducible in testing (data extraction: test-retest)

Implementation	• Baseline and potential change in baseline;evidence of sensitivity to change (data extraction);
	• Exception reporting/gaming (interviews with practice staff);
	• Changes in practice organisation; potential barriers; workload (interviews with practice staff and workload diaries)
	• Unintended consequences (interviews with practice staff)

Changes to any existing QOF indicators	• Summary of any suggested changes to existing QOF indicators as a result of piloted indicators

Changes in wording of the indicator(s)	• Summary of any suggested changes to indicator wording

Cost effectiveness	• Summary of evidence of cost effectiveness (cost-effectiveness modelling)

**Overall recommendation**	1) no major barriers/risks/issues/uncertainties
	2) some barriers/risks/issues/uncertainties but okay
	3) major barriers/risks/issues/uncertainties preclude it

Summary feedback table of indicator testing protocol

*RAND/UCLA Appropriateness Method overall panel rating of ≥ 7 with agreement (80% of panellist's ratings within the 3-point tertile of the overall median)

#### Clarity and necessity: content validity

The ratings from the RAND/UCLA Appropriateness Method are used for the assessment of the clarity and necessity of the indicators (proxies for content validity).

#### Technical feasibility and reliability

The assessments of technical feasibility and reliability are akin to the NCQA attributes of feasibility and scientific soundness [6] and the AMA-PCIP attributes of feasibility and reliability [9] and are carried out in conjunction with the UK National Health Service Information Centre (NHSIC). Feasibility relates to evidence about whether accurate data are available and collectable in current family practice information systems, and supported by current methods of data extraction for QOF. The assessment of reliability focuses on quality assuring reproducibility. The Information Centre writes a specification for each indicator, detailing how data will be extracted from patient electronic medical records. Piloting enables these data extraction rules to be tested and for any errors to be identified and rectified.

#### Implementation - workload

Within each participating practice, we ask the staff most involved in the pilot and the day-to-day engagement with QOF to complete workload diaries. This enables us to assess the effort associated with implementing pilot indicators and also feeds in to subsequent cost-effectiveness analyses. The diaries include sections on the date and time of activity, which indicator the activity related to, activity undertaken using a tick box of options (i.e. computer query searching, meetings, clinical appointments etc), and whether the activity was scheduled or involved not doing other planned work. (These diaries are available on request).

#### Implementation: sensitivity to change and discrimination/variance

We extract data from the family practices' electronic medical record systems for each indicator using the data extraction specification rules at the start (baseline) and at the end of the pilot. Obtaining both sets of data enable tracking of performance (sensitivity to change) and cost-effectiveness modelling to be undertaken. Obtaining baseline and final data, the testing protocol also enables recommendations about where to set potential payment thresholds.

#### Acceptability and implementation

We assess the acceptability and implementation of indicators through semi-structured interviews with doctors, nurses and administrative staff involved in piloting. Assessment of issues and barriers arising from implementation is a key aspect also of the NCQA [6], QUALIFY [7] and AMA-PCIP [9] testing protocols respectively.

#### Cost effectiveness

Cost effectiveness considers whether the costs associated with an indicator are outweighed by the benefits accrued by the health service. The approach we use to evaluate the economic implications of indicators addresses two issues. The first determines whether the activity or intervention is cost effective and would result in benefits which are greater than the costs of undertaking the activity. Health benefits are measured in Quality Adjusted Life Years (QALYs) [23] which are valued in monetary terms at £25,000 (€29,071) each. This is the mid-point of the implicit cost effectiveness threshold of £20,000-£30,000 (€23,259 -€34,889) per QALY gained, which has been adopted by NICE [24]. The net benefit calculation subtracts the delivery costs of implementation and the payments from the monetarised health benefits: Net benefit = (monetised benefit - delivery cost) - QOF payment. The second relates to the level of payments that can be economically justified to increase levels of desired activities whilst retaining net benefits to the health service. This is done by hypothesising a link with improved patient outcomes but is possible only where robust evidence to support the hypothesis was available, which is more likely for indicators which have a direct therapeutic effect [25]. In reality, this evidence is often not available.

##### Indicator piloting: data collection methods and analyses

We have recruited four separate cohorts of 30 representative family practices across England from twelve Primary Care Trusts, the current organisational units with the English NHS of approximately 300,000 patients, to pilot the clinical indicators. Each pilot cohort includes practices with different clinical information systems, and practices which are nationally representative in terms of size, QOF achievement and Index of Multiple Deprivation (IMD). Each cohort of practices pilots indicators for six months on a rolling basis.

#### Overall recommendations

Based on the evidence obtained from piloting, we make a recommendation for each indicator on the four key attributes of acceptability, feasibility, reliability and implementation (Table 1). Evidence for clarity and content validity are based on the fact that indicators are underpinned by a NICE/SIGN guideline and RAND/UCLA Appropriateness Method ratings.

The three potential recommendations from piloting are:

1) There are no major barriers/risks/issues/uncertainties.

2) There are some barriers/risks/issues/uncertainties but these can be addressed before national roll out.

3) There are major barriers/risks/issues/uncertainties that preclude the indicators from recommendation for its inclusion in QOF.

We then use these recommendations as the basis for an overall recommendation (Table 1). These recommendations are then considered by the NICE Advisory Committee which makes decisions about which indicators have passed piloting, these decisions are then validated by NICE (Figure 1). They will then be submitted subsequently for negotiations between the British Medical Association General Practitioners Committee and NHS Employers for possible inclusion in QOF. It is at this stage that the process of assigning points and payments to tested and validated indicators takes place.

The study had full ethical committee approval (North West 4 Research Ethics Committee: Liverpool North: 09/H1001/74).

## Results

In June 2009 the NICE Advisory Committee recommended six areas for indicator development (http://www.nice.org.uk/media/F3E/8C/QOFIACMinutesJune2009.pdf) to be piloted in pilot 1 (October 2009-March 2010): asthma, dementia, diabetes, myocardial infarction, serious mental illness and palliative care.

### Clarity and necessity: content validity

We rated the clarity and necessity of the indicators using the RAND/UCLA Appropriateness Method. Thirteen indicators were rated clear and necessary and therefore went forward to piloting (Table 2).

**Table 2:** Indicators piloted and examples of issues raised as a result of piloting

	Clinical area	Indicator wording	Implementation issues raised as a result of piloting	Recommendations
1	Asthma	The percentage of patients with asthma who have had an asthma review in the previous 15 months that includes an assessment of asthma control using the 3 Royal College of Physicians questions (control of daytime, night time and activity limiting symptoms in the last week.	• Technical feasibility: need for revised IT data entry templates	Acceptability: no barriers
			• Administration of questions by phone, post or face to face	Feasibility: some barriers
				Reliability: some barriers
				Implementation: no barriers
				Overall: some barriers to address

2	Dementia	The percentage of patients with a new diagnosis of dementia to have FBC, calcium, glucose, renal and liver function, thyroid function tests, serum vitamin B12 and folate levels recorded 6 months before or after entering on to the register	• Inconsistency with dementia referral pathways/referral governance	Acceptability: no barriers
			• Variation in practice - QOF danger of standardizing practice	Feasibility: no barriers
			• Low prevalence in some practices (as confirmed by the pilot)	Reliability: no barriers
			• Tests of calcium levels not always routinely done	Implementation: some barriers
				Overall: some barriers to address but can go forward

3	Diabetes	The percentage of patients with diabetes with a record of testing of foot sensation using a 10 g monofilament or vibration (using biothesiometer or calibrated tuning fork), within the preceding 15 months	• Changes required to data entry diabetes templates where not already included	Acceptability: no barriers
			• Education or training of nurses in some practices.	Feasibility: no barriers
				Reliability: no barriers
				Implementation: no barriers
				Overall: no barriers

4	Diabetes	The percentage of patients with diabetes with a record of a foot examination and risk classification: 1) low risk (normal sensation, palpable pulses), 2) increased risk (neuropathy or absent pulses), 3) high risk (neuropathy or absent pulses plus deformity or skin changes or previous ulcer) or 4) ulcerated foot within the preceding 15 months	• Changes required to data entry diabetes templates where not already included	Acceptability: no barriers
			• Education or training of nurses in some practices.	Feasibility: no barriers
			• Routine data recording	Reliability: no barriers
			• Workload implications for practice staff	Implementation: some barriers
			• Attribution problems in terms of payments attached to QOF if performed outside the practice	Overall: some barriers to address but can go forward
			• Time taken to perform	

5	Myocardial Infarction	The percentage of patients with a history of myocardial infarction (from 1 April 2011 {from 1 October 2009 for the purposes of piloting} currently treated with an ACE inhibitor, aspirin or an alternative anti-platelet therapy, beta-blocker and statin (unless a contraindication or side effects are recorded)	Implementation issues:	Acceptability: some barriers
			• Problems of attribution/hospital led prescribing	Feasibility: no barriers
			• PCT formulas/guidance/pharmacy advisors content and advice is contrary to NICE guidelines	Reliability: no barriers
			MI was the only area for which there is useable cost-effectiveness data	Implementation: some barriers
				Overall: some barriers to address but can go forward

6	Myocardial Infarction	The percentage of patients with a history of myocardial infarction who have a record of intolerance or allergy to an ACE inhibitor who are currently treated with an ARB (unless a contraindication or side effects are recorded)	• Conflicts with variations in local guidance (in some areas PCT stipulates trying 3 ACEs before an ARB, in others ARB is front line treatment)	Acceptability: some barriers
			• Variation in percentage of patients on ARB	Feasibility: no barriers
			• Problems of attribution/hospital led prescribing	Reliability: no barriers
			• Variations in local procedures in i.e. PCT formulas/guidance/pharmacy advisors	Implementation: some barriers
			• Private initiated prescribing effects % of patients on ARB	Overall: some barriers to address but can go forward
			MI is the only area for which there is useable cost-effectiveness data	

7	Serious Mental Illness	The percentage of patients with schizophrenia, bipolar affective disorder and other psychoses who have a record of alcohol consumption in the preceding 15 months	• Seldom heard group, whom often consult opportunistically. Difficulty getting patients to come back in	Acceptability: some barriers
				Feasibility: no barriers
				Reliability: no barriers
				Implementation: some barriers
		
8	Serious Mental Illness	The percentage of patients with schizophrenia, bipolar affective disorder and other psychoses who have a record of BMI in the preceding 15 months	• Low levels of cervical screening	Overall: some barriers to address but can go forward
		
9	Serious Mental Illness	The percentage of patients with schizophrenia, bipolar affective disorder and other psychoses who have a record of blood pressure in the preceding 15 months	• Perceived clinical irrelevance to younger patients re. annual cholesterol, BP, HBA1c/glucose check: these are not routinely done in all practices for all patients on the current register	
		
10	Serious Mental Illness	The percentage of patients with schizophrenia, bipolar affective disorder and other psychoses who have a record of total cholesterol: hdl ratio level in the preceding 15 months	• May create undue focus on individual processes/indicators in unbundled indicators rather than the overall physical health of patient "appropriate to their age, gender and health status"	
		
11	Serious Mental Illness	The percentage of patients with schizophrenia, bipolar affective disorder and other psychoses who have a record of blood glucose level or HBA1c in the preceding 15 months		
		
12	Serious Mental Illness	The percentage of women aged 25-64 with schizophrenia, bipolar affective disorder and other psychoses who have a record of cervical screening within the last 5 years		

13	Palliative Care	The percentage of patients on the palliative care register who have a preferred place to receive end-of-life care documented in the records	• Perceived potential harm to patients	Acceptability: preclude
			• Changes to timing of which patients are put on the register: the palliative care register is perceived to often be quite subjective	Feasibility: no barriers
			• The indicator does not pro-actively encourage GPs to keep the preferred place for end of care up to date	Reliability: no barriers
			• Anxiety over the rigidity of the stipulated timeframes which are too prescriptive	Implementation: preclude
			• Undue focus on one isolated question from a mutlifaceted and complex issue	Overall: preclude
			• Problems of attribution/not necessarily general practice team's responsibility	

Examples of issues raised for each of these piloted indicators in terms of the other key attributes of the testing protocol (technical feasibility and reliability, acceptability and implementation) and the overall recommendations for each indicator in pilot 1 are summarised in Table 2.

### Technical feasibility and reliability

During the pilot, we found data measurement specifications relating to the asthma, diabetes and dementia indicators (Table 2). For example, problems were identified for the asthma indicator in relation to the reliability of extracting data when data for daytime, night-time and activity limiting symptoms were recorded in the patient's medical record on the same or different days.

### Implementation - workload

The majority of workload diaries showed that most of the QOF indicator work was carried out by practice nurses. The diaries enabled us to quantify how much time was spent on each component activity including face to face work with patients and also underpinning administrative work. The majority of GP time during the piloting process was spent in team meeting (73%) and in addressing the dementia and palliative care indicators, whereas the majority of nurse time spent on piloting was spent in consultations with patients (90%) with asthma, diabetes or myocardial infarction. The care associated with piloting the serious mental illness indicators was divided between the doctors and nurses.

### Implementation: sensitivity to change and discrimination/variance

Data for the myocardial infarction four drugs indicator (Aspirin, Betablocker, Statin and ACE inhibitor) showed low levels of achievement at baseline, making it a good candidate for evidence based quality improvement and therefore financial incentivization.

### Acceptability and implementation

We conducted semi-structured interviews with 57 members of staff in 24 family practices: 21 family doctors, 16 practice managers, 12 nurses and eight others (mostly information technology experts) during April-May 2010.

All participants in Pilot 1 thought that piloting indicators was essential. The value of piloting was seen as akin to a 'reality check', and learning process highlighting potential problems which could then be addressed prior to the indicator being implemented on a national level.

#### Issues raised by piloting

Examples of issues raised by participants as a direct result of piloting are shown in Table 2. These included identifying indicators with potential harm to patients (e.g. the palliative care indicator), low numbers of eligible patients (e.g. dementia, myocardial infarction), and a need for changes in practice organization such as changes to computer templates and workload implications such as time to carry out the incentivised aspect of care if not already routinely provided (e.g. diabetes foot risk assessment). In addition, participants identified indicators with conflicting hospital and primary care prescribing patterns; for example, where patients were discharged from hospital after a myocardial infarction with prescriptions for drugs that differed from the drugs stipulated in the myocardial infarction indicator.

### Cost effectiveness

Cost effectiveness analyses of the piloted indicators showed that only the myocardial infarction indicators could be linked directly to health gains, because there was clear clinical and economic evidence that recommended all patients presenting with an acute myocardial infarction should be treated, unless contraindications apply, with an ACE inhibitor, aspirin and beta-blockers indefinitely [26,27]. However, for the remaining indicators there were insufficient data to make a clear link with health benefits, making cost-effectiveness analysis speculative.

### Overall recommendations

Based on the evidence obtained from piloting, twelve indicators passed the indicator testing protocol. The palliative care indicator was recommended for rejection because, while technically feasible and reliable, it was unacceptable to practice staff and raised concerns about upsetting the balance of the doctor patient relationship and potentially causing actual patient harm due to the lack of sensitivity of a single isolated target approach. Piloting also identified a number of implementation issues and unintended consequences that were subsequently rectified or removed prior to national roll out for the other 5 piloted areas (Table 2).

## Discussion

### Strengths and limitations of the testing protocol

#### Strength

In this paper we have described the indicator testing protocol for assessing potential new QOF indicators using 'real time' examples from the first pilot (October 2009-March 2010). The testing protocol identified a number of implementation issues and unintended consequences associated with piloted indicators. It demonstrates the value of piloting as a prerequisite for all policy-makers prior to introducing indicators as part of quality assessment. The palliative care indicator is a particular exemplar of the value of a multiple attribute indicator testing protocol - while technically feasible and reliable, it had poor acceptability and was rejected for possible inclusion in QOF.

#### Limitations

This testing protocol has a number of possible limitations. For example, a cohort of practices involves working with a maximum of 30 pilot family practices although these are explicitly selected to be nationally representative. Second, the piloting period, by necessity due to tight deadlines in the overall process, is only six months compared to 12 months in the HEDIS piloting process. A longer piloting period would for each Pilot would enable potential changes to bed in and practice staff more time to engage with the indicators. Third, the first pilot confirmed that there are currently little data for cost effectiveness modelling and that cost-benefit analysis is problematic for process measures because of the difficulty of linking process to outcome. Fourth, the piloting process tests the effect of piloted indicators but it provides no information about how practices respond to a given financial incentive for a quality indictor as the process of assigning points and financial rewards for validated indicators takes place as part of subsequent negotiations between the NHS Employers and BMA/GPC.

#### Implications for practice and policy

In this paper, we have described a testing protocol that is used to assess potential indicators for the UK QOF. However, whilst our testing protocol may act as a prototype, it is important that each country takes account of its local context. There are real dangers in directly translating indicators across settings [28,29]. For example, countries such as the United States [2] or Australia [30] face significant technical and institutional obstacles in introducing family practice indicator based incentives schemes because of the availability and comprehensiveness of clinical data systems. Moreover, the UK QOF is underpinned by the fact that patients register with a single family practice, unlike many other countries, enabling practice registers of patients with given conditions to be created.

We suggest that indicators require empirical evidence of testing for key attributes such as acceptability and feasibility before they can be safely used [5,6,31,32]. There are common attributes shared by indicator testing protocols (Table 3). However, while the US HEDIS methodology and QUALIFY [7] focus on the three key concepts of relevance, scientific soundness and feasibility,[6] the AMA-PCIP protocol focuses on a wider range of attributes over the short and long term [9]. In the US agencies such as The National Quality Forum (NQF) [33] and the National Quality Measure Clearinghouse (NQMC) [34] have also proposed measure standards but these have not been developed into a measure testing protocol.

**Table 3:** Attributes common to published indicator testing protocols

UK-ITP^+^	**AMA-PCIP **[10]	**NCQA/HEDIS **[6]	**Qualify **[7]
NICE prioritisation	Needs assessment	Relevance	Relevance

Acceptability	Acceptability		

Clarity	Clarity	Precision =	Clarity/specificity

Feasibility	Feasibility	Feasibility	Feasibility

Reliability	Reliability	Reproducibility~	Reliability~

Validity *	Validity *#	Validity *	Validity *~

Implementation	Implementation	Implementation	Implementation
-Unintended	-Unintended	-Controllability¬	-Risks/side effects¬
Consequences	Consequences	-Benchmarking/	-Benchmarking/
-Benchmarking/	-Benchmarking		
Sensitivity to change -Workload			

Cost effectiveness		Cost effectiveness¬	

^+ ^UK-ITP: Indicator testing protocol described in this paper

Validity *: face and content validity underpinned by evidence based guideline

Validity#: predictive validity

~ As part of *Scientific Soundness*

¬ As part of *Relevance*

^= ^As part of *Feasibility*

The indicator testing protocol we are advocating in this paper codifies existing experience in this area and proposes a testing protocol using a 6 month piloting timeframe. It privileges implementation issues but places the views of coal face practice staff at the centre of the piloting process. The worth of a test piloting protocol is the overall sum of its parts. The palliative care indicator reported in this paper provides an exemplar of the importance of a multiple attribute indicator testing protocol.

## Conclusions

The UK government currently spends over 1 billion pounds each year on QOF. Each UK pilot cost £150,000 (0.0005% of the overall cost). The act of piloting indicators is therefore value for money as it identifies implementation issues of acceptability and unintended consequences as well as technical reliability and feasibility that can be addressed and rectified prior to national roll out. Moreover, it highlights indicators that should not be included.

An indicator testing protocol must act as a foundation stone for the field testing and development of country specific quality indicators for pay-for-performance or quality improvement schemes. Local adaptations of this protocol could be used by policy-makers and researchers to empirically test the likely effect of implementing indicators. Whilst national in scope, the lessons are, we hope, therefore generalizable for an international audience.

## Competing interests

At the time Pilot 1 was conducted, Stephen Campbell and Helen Lester were contracted to the National Institute for Health and Clinical Excellence to provide advice on removal of indicators and pilot new indicators for the Quality and Outcomes Framework. The views expressed are those of the authors and do not necessarily represent the views of NICE or its independent Quality and Outcomes Framework advisory committee.

http://www.nice.org.uk/media/742/32/QOFProcessGuide.pdf

## Authors' contributions

SC and HL conceived and designed the study. All Authors wrote and reviewed the manuscript and read and approved the final manuscript.

## Appendix 1: Summary of indicator testing protocol

### Clarity

• The indicator wording is clear and precise with unambiguous language that reflects a specific domain of content, as rated by the RAND/UCLA Appropriateness Method

• The indicator is within the control of the clinician/practice, as rated by the RAND Appropriateness Method

### Content validity

• The indicator statement represents high quality care and is therefore a valid indicator of quality. There is sufficient evidence/professional consensus to support it and there are clear benefits to the patient receiving the care (or the benefits significantly outweigh the risks).

◦ Each indicator is underpinned by a NICE/SIGN guideline.

◦ Each indicator is rated necessary by a RAND Appropriateness Method, which is based on physicians/practice staff adhering to the indicator providing a higher quality of care/service than those who are not doing so.

### Technical feasibility and reliability of data extraction

• Ability to write and integrate data extraction specifications into health information systems from all family practices

• Ability to generate reproducible test reports within a reasonable time frame and budget from all family practices

### Acceptability

• Alignment to professional values and family practice

• Likely patient benefit

### Implementation

• Discriminate validity: assessment of indicator to discriminate within a nationally representative sample of family practices

• Sensitivity to change: assessment of current baseline of the indicator and potential change in baseline at the end of piloting

• Clinical practice staff are able to interpret the indicator

• Potential for *gaming/manipulation *(exception reporting)

• Changes required in practice organization to implement the indicator (i.e. acquisition and/or modification of IT; changes in physical capital or staffing; changes to practice policies and culture).

• Workload implications of implementing the indicator

• Potential barriers to the implementation of the indicators

• Unintended consequences to the implementation of the indicator: these can be positive or negative in nature (i.e. disruption to clinical or organisational workflow, 'spillovers' that may be negative (diversion of effort) or positive (encouraging general quality improvement).

## Pre-publication history

The pre-publication history for this paper can be accessed here:

http://www.biomedcentral.com/1471-2296/12/85/prepub
